# Association Between Spinal Alignment and Trunk and Lower Extremity Muscle Mass in Preoperative Total Hip Arthroplasty: A Cross-Sectional Analysis

**DOI:** 10.7759/cureus.88379

**Published:** 2025-07-20

**Authors:** Shuro Furuichi, Atsushi Shinonaga, Shigeru Mitani, Mana Uekawa, Saki Kakeya, Hiromi Matsumoto

**Affiliations:** 1 Department of Bone and Joint Surgery, Kawasaki Medical School, Okayama, JPN; 2 Rehabilitation Center, Kawasaki Geriatric Medical Center, Okayama, JPN; 3 Rehabilitation Center, Kawasaki Medical School Hospital, Kurashiki, JPN; 4 Kawasaki Medical School Hospital, Kurashiki, JPN; 5 Department of Physical Therapy, Faculty of Rehabilitation, Kawasaki University of Medical Welfare, Matsushima, JPN

**Keywords:** muscle volume, sagittal spino-pelvic alignment, sarcopenia, total hip arthroplasty, trunk balance

## Abstract

Background

The sagittal vertical axis (SVA) reflects trunk balance. This study investigated the relationship between trunk balance and muscle mass, as well as the prevalence of sarcopenia in preoperative total hip arthroplasty (THA) patients using SVA.

Materials and methods

This retrospective cohort study included 126 patients who underwent THA over a one-year period and met the eligibility criteria. Data collected included body mass index, duration of illness, number of falls, sarcopenia, sagittal alignment, pelvic tilt, and skeletal muscle mass. Patients were divided into two groups based on SVA: the unbalanced (U) group (SVA ≥50 mm) and the balanced (N) group (SVA <50 mm).

Results

The U group and the N group had n=61 and n=65, respectively. There were no significant differences in disease duration (9.3 ± 13 vs. 10.8 ± 13.3 months) or prevalence of sarcopenia (14 (23%) vs. 14 (22%)) between the U and N groups. However, the nonoperative lower extremity muscle mass was significantly lower in the U group (5.7 ± 1.2 kg) compared to the N group (6.1 ± 1.3 kg; *p* = 0.04). Trunk muscle mass was also lower in the U group (17.1 ± 3.2 kg vs. 18.4 ± 3.4 kg; *p* = 0.026). Pelvic-spine alignment angles (sacral slope (SS)) were significantly smaller in the U group in the standing (35.4° ± 13.1° vs. 40.6° ± 10.6°), supine (7.4° ± 15.7° vs. 14.3° ± 13.3°), and seated (36.2° ± 12° vs. 41.3° ± 10°) positions.

Conclusion

SVA is associated with trunk balance in preoperative THA patients. Those with poor trunk balance (SVA ≥50 mm) exhibited decreased muscle mass in the trunk and lower limbs on the nonoperative side, highlighting the importance of assessing trunk balance using SVA in this population.

## Introduction

The sagittal vertical axis (SVA), defined as the distance between the C7 plumb line and the posterior border of the sacrum, is a key parameter of spinal alignment [1,2]. SVA reflects trunk balance and has been widely used as an important index in spine surgery. The relationship between SVA and muscle mass has been reported in conditions such as lumbar spondylolisthesis [3-5]. However, this association has not been reported in patients with hip osteoarthritis (OA).

Understanding this relationship may allow prediction of muscle condition based on trunk balance. SVA also significantly impacts quality of life [6], emphasizing the importance of evaluating alignment. Several studies, including randomized controlled trials, have shown the benefits of preoperative exercise in hip OA [7,8]. An SVA of 50 mm or more indicates poor trunk balance [9].

Maintaining trunk balance in hip OA is essential for both quality of life and preserving lower extremity and trunk muscle mass. Alterations in spinal alignment after corrective spine surgery can impair balance and increase the risk of hip dislocation [10-12]. Understanding the muscle mass needed to maintain trunk balance is therefore important in preventing postoperative dislocation - a key complication in total hip arthroplasty (THA).

Sarcopenia, marked by reduced muscle mass [13,14], may contribute to imbalance. Investigating the relationship between muscle mass and trunk balance is thus clinically important for preventing complications. Complications may include dislocation due to malalignment, spinal compression fractures, and postoperative back and thigh pain. This cross-sectional study aimed to examine whether spinal alignment and pelvic tilt are associated with skeletal muscle mass in patients undergoing THA.

## Materials and methods

We included 264 patients who underwent their first THA at our hospital over a one-year period from January to December 2022. Exclusion criteria were multinational status, lack of consent, and age under 40 years. In cases of bilateral THA, the most recent surgery was defined as the operative side.

This study is part of the K-HIP study, a clinical research project initiated in 2022 that aims to identify and address specific issues in patients undergoing THA. The project approaches these patients from multiple perspectives to enhance understanding and improve outcomes.

Survey measurements

The study items included body mass index (BMI), duration of disease (months), cases of falls in the past year, presence of sarcopenia, and muscle mass of the lower limbs and trunk (kg). Muscle mass was measured using a body composition analyzer (InBody 770, InBody Japan, Tokyo, Japan), which provided values for lower limb skeletal muscle mass on the operated side (kg), lower limb skeletal muscle mass on the nonoperated side (kg), and trunk muscle mass (kg). Patients themselves were surveyed in the form of a questionnaire. They were asked whether they had experienced any falls in the past year.

The measurement is made by applying a weak electric current using a method called bioelectrical impedance analysis (BIA). While metallic implants such as artificial joints may theoretically influence the results, previous studies have reported that their impact on BIA measurements is minimal. Therefore, we consider the influence of the implant to be negligible. As evidence, a recent article by Ukai and Watanabe (2023) confirmed that total hip arthroplasty implants do not significantly alter BIA measurements [15].

Sarcopenia was assessed based on the diagnostic criteria revised by the Asian Working Group for Sarcopenia in 2019 [16]. The criteria included: grip strength <28 kg for men and <18 kg for women; walking speed <1.0 m/s; and skeletal muscle index <7.0 kg/m2 for men and <5.7 kg/m2 for women. We defined sarcopenia as the presence of both muscle function (grip strength and walking speed) and low muscle mass. Both were assessed on the day of admission and the day before surgery.The walking speed is the normal walking speed, and the gait speed is calculated by walking 10 meters (twice on a flat 10-metre path).

**Figure 1:**
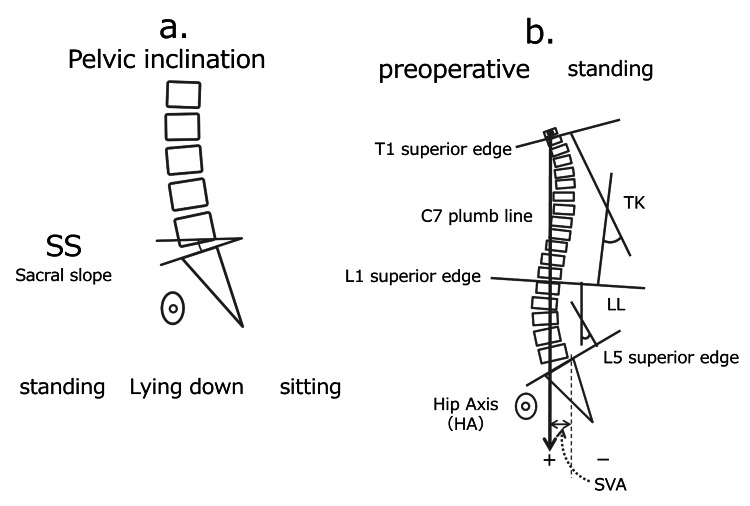
Pelvic Tilt and Spinal Alignment Measurements in Different Postures (a) Pelvic tilt (sacral slope (SS) standing, sitting, and lying), (b) Spinal alignment (Thoracic kyphosis (TK), lumbar lordosis (LL), sagittal vertical axis (SVA)). Image Credit: Shuro Furuichi

Lumbar lordosis (LL) and thoracic kyphosis (TK) were measured as indicators of spinal alignment using 10plain radiographs. Pelvic incidence (PI) and sacral slope (SS) were assessed as pelvic morphology angles. All parameters were initially measured in the standing position. LL was defined as the angle between the superior margins of L1 and S1, reflecting the degree of lumbar curvature. Additionally, SS and LL were measured in the seated and supine positions (Figure 1). The PI-LL mismatch in the standing position was also evaluated to assess the trunk balance between the pelvis and spine. Spinal alignment was defined based on SVA: an SVA >50 mm was defined as unbalanced (U group), and an SVA <50 mm as normal (N group), based on the report by Schwab et al. [9]. X-rays were taken during an outpatient visit two weeks immediately prior to surgery.

Ethics statement

This study was approved by the Ethics Committee of Kawasaki Medical School (Ethics No. 5515-00). Patients were provided the option to discontinue the study via our website. Due to the noninvasive and retrospective nature, the Ethics Committee waived the requirement for written informed consent from each participant.

Statistical analysis

Descriptive analyses were conducted using mean ± standard deviation (SD) for continuous variables and frequency (%) for categorical variables. Independent-samples t-test was used to compare continuous variables, including each parameter, while the chi-square test was applied for categorical variables. Logistic regression analysis was performed using SVA ≥50 mm as the explanatory variable (i.e., U group versus N group). The dependent variables were skeletal muscle mass of the operated leg, skeletal muscle mass of the trunk, and skeletal muscle mass of the non-operated leg, as well as age, gender, BMI, and duration of disease. Receiver operating characteristic (ROC) analysis was conducted to evaluate the discriminative ability of muscle mass in relation to alignment, with sensitivity, specificity, and area under the curve (AUC) calculated. All statistical analyses were performed using Stat Flex (Version 7, Artech Co., Ltd., Osaka, Japan; http://www.statflex.net). A p-value of less than 0.05 was considered statistically significant.

## Results

Of the total 264 cases, 235 were included in the study after excluding 29 cases. The excluded cases comprised two patients from other countries, 21 nonconsenting patients, and six patients under 40 years of age. Additionally, cases lacking preoperative radiographs or skeletal muscle mass measurements were excluded due to missing data. The number of patients with missing measurements was 109.

The mean age at surgery was 66.9 years (range: 41-91 years). Of the included patients, 102 underwent unilateral THA, and 24 underwent bilateral THA. The primary diagnosis included 115 patients with hip OA, 11 with idiopathic osteonecrosis of the femoral head (ION), and four with a history of compression fracture.

**Figure 2:**
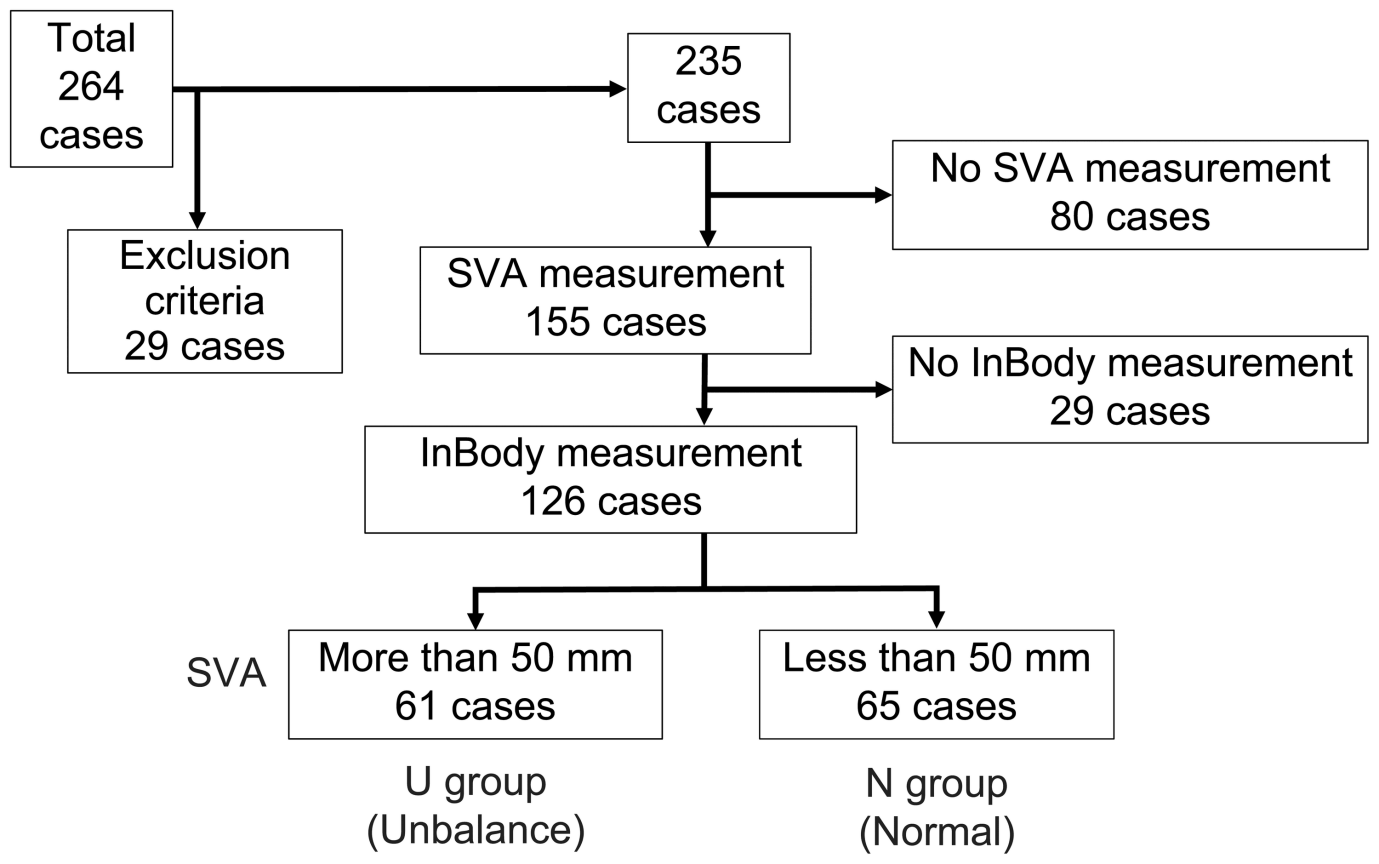
Flowchart of the case selection SVA: sagittal vertical axis

From the original 264 patients, 235 met the eligibility criteria. Of these, 126 patients were further included in the final analysis after excluding those without available SVA and InBody measurements. The final cohort comprised 61 patients in the U group and 65 in the N group. A flowchart of patient inclusion is shown in Figure 2.

The mean BMI of the 126 patients was 24.7 ± 4.5 kg/m2, the mean duration of disease was 10.1 ± 13.2 years, and the reported number of falls per year included one fall in 24 patients and two falls in 14 patients (Table 1). The SS averaged 38.8° ± 11.5°, standing LL was 40.8° ± 16.7°, sitting LL was 18.9° ± 15.3°, and supine LL was 38.4° ± 16.4° (Table 1). TK averaged 30.1° ± 12.8°, and the SVA averaged 50.5 ± 37.5 mm. Skeletal muscle mass was 5.7 ± 1.3 kg for the operative leg, 5.9 ± 1.3 kg for the nonoperative leg, and 17.8 ± 3.4 kg for the trunk (Table 1).

**Table 1:** Baseline characteristics and radiographic measurements of preoperative total hip arthroplasty (THA) patients (n = 126) BMI: Body Mass Index; SS: Sacral Slope; LL: Lumbar Lordosis; TK: Thoracic Kyphosis; SVA: Sagittal Vertical Axis; PI: Pelvic Incidence; PI-LL: Pelvic Incidence minus lumbar lordosis. Muscle mass measurements are expressed in kilograms (kg). Data are presented as mean ± standard deviation unless otherwise specified.

Variables	Values
Age (years)	66.3 ± 9.3
BMI (kg/m^2^)	24.7 ± 3.9
duration of disease (year)	10.1 ± 13.2
Cases of falls in the past year	38 cases (30%)
Sarcopenia	28 cases (22%)
Radiographic parameters	
SS (standing)	38.1 ± 12.2
SS (sitting)	11.0 ± 14.9
SS (lying down)	38.8 ± 11.5
LL(standing)	40.8 ± 16.7
LL(sitting)	18.9 ± 15.3
LL(lying down)	38.4 ± 16.4
TK	30.1 ± 12.8
SVA	50.53 ± 37.5
PI	53.0 ± 14.6
PI-LL	14.6 ± 15.3
Muscle mass	
Surgical leg skeletal muscle mass (kg)	5.7 ± 1.3
Nonoperative leg skeletal muscle mass (kg)	5.9 ± 1.3
Trunk skeletal muscle mass (kg)	17.8 ± 3.4

In the comparison between the U and N groups, 14 patients (23%) in the U group and 14 patients (22%) in the N group had sarcopenia (χ2 test, p = 1.00), indicating no significant differences between the groups in terms of BMI, duration of illness, or history of falls (Table 2).

**Table 2:** Comparison of baseline characteristics between poor balance (U group) and normal balance (N group)) Independent-samples t-test *p < 0.05; **Chi-square test

Variable	U group (n = 61)	N group (n = 65)	Test statistic	p-value
Age (years)	69.5 ± 9.0	63.2 ± 8.6	t = −4.03	*0.04
BMI(kg/m^2^)	24.7 ± 3.0	24.7 ± 4.5	t = 0.1	0.18
Disease duration(year)	9.3 ± 13.0	10.8 ± 13.3	t = 0.63	0.57
Cases of at least one fall per year.	21 (34%)	17 (26%)	χ^2^ = 0.66	^**^0.41
Sarcopenia	14 (23%)	14 (22%)	χ^2^ = 0.00	^**^1.00

Regarding X-ray parameters, SS was significantly smaller in the U group in all positions, indicating a more posterior pelvis tilt: standing SS (p = 0.02), sitting SS (p = 0.01), and lying SS (p = 0.006). This suggests that patients with poor spinal balance exhibited posterior pelvic tilt across all postures. Lumbar kyphosis was also significantly reduced in the U group, reflecting increased lumbar kyphosis (Table 3).

**Table 3:** Comparison of pelvic tilt and spinal alignment parameters between U and N groups SS: Sacral Slope; LL: Lumbar Lordosis; TK: Thoracic Kyphosis; SVA: Sagittal Vertical Axis; PI: Pelvic Incidence; PI-LL: Pelvic Incidence minus lumbar lordosis. Values are presented as mean ± standard deviation. Comparisons between grSS: Sacral Slope; LL: Lumbar Lordosis; TK: Thoracic Kyphosis; SVA: Sagittal Vertical Axis; PI: Pelvic Incidence; PI-LL: Pelvic Incidence minus lumbar lordosis. Values are presented as mean ± standard deviation. Comparisons between groups were performed using the Independent-samples t-test. *p < 0.05 was considered statistically significant.oups were performed using the Independent-samples t-test. *p < 0.05 was considered statistically significant.

N = 126	U group n = 61	N group n = 65	Test statistic	p-value
SS (standing)	35.4 ± 13.1	40.6 ± 10.6	t = 2.44	*0.02
SS (sitting)	7.4 ± 15.7	14.3 ± 13.3	t = 0.01	*0.01
SS (lying down)	36.2 ± 12.0	41.3 ± 10.0	t = 2.75	*0.006
LL (standing)	37.4 ± 17.8	44.0 ± 15.0	t = 2.22	*0.03
LL (sitting)	17.2 ± 14.0	20.5 ± 16.3	t = 1.21	0.22
LL (lying down)	37.3 ± 17.7	39.3 ± 15.0	t = 0.731	0.47
TK	29.6 ± 13.2	30.6 ± 12.3	t = 0.46	0.64
SVA	81.4 ± 26.0	21.6 ± 19.2	t = −14.65	0.000
PI	54.8 ± 14.1	56.1 ± 12.2	t = 0.55	0.30
PI-LL	17.3 ± 15.6	12.1 ± 14.5	t = −1.94	*0.04

There was no significant difference in the skeletal muscle mass of the operative side between the two groups. However, the U group had significantly lower skeletal muscle mass in the nonoperative lower limb (p = 0.04) and in the trunk (p = 0.03) (Table 4).

**Table 4:** Comparison of muscle mass by site *p < 0.05, Independent-samples t-test

Variables	U group (n = 61)	N group (n = 65)	Test statistic	p-value
Skeletal muscle mass of the surgical leg(kg)	5.6 ± 1.2	5.9 ± 1.4	t = 1.58	0.11
Nonoperative lower extremity skeletal muscle mass(kg)	5.7 ± 1.2	6.1 ± 1.3	t = 2.00	*0.04
Trunk skeletal muscle mass(kg)	17.1 ± 3.2	18.4 ± 3.4	t = 2.25	*0.03

**Figure 3:**
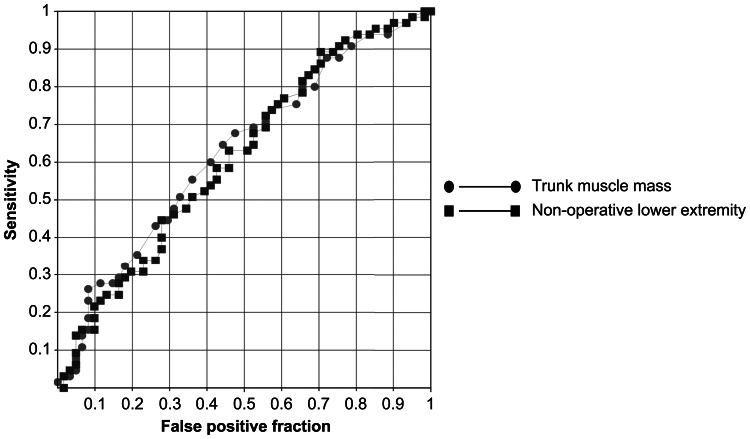
Receiver operating characteristic (ROC) curves for nonoperative lower extremity skeletal muscle mass and trunk muscle mass

ROC curve analysis revealed a cutoff value of 5.617 kg for the nonoperative lower limb muscle mass, with both sensitivity and specificity of 55.3%, and an AUC of 0.6116 (standard error of 0.05). For trunk skeletal muscle mass, the cutoff value was 17.0 kg, also with a sensitivity and specificity of 55.3%, and an AUC of 0.63 (standard error of 0.05) (Figure 3, Table 5).

**Table 5:** The results of multivariate logistic regression analysis The results of multivariate logistic regression analysis with sagittal imbalance as the dependent variable and each item as the independent variable. *p < 0.05, Independent-samples t-test

Variable	Coefficient (β)	Standard error	p-value	95% CI for β
Intercept	-5.24	2.871	0.068	[-10.87–0.39]
Trunk muscle mass (kg)	-0.152	0.171	0.375	[-0.49–0.18]
Non-operative leg mass (kg)	-0.015	0.713	0.983	[-1.41–1.38]
Operative leg mass (kg)	0.145	0.624	0.816	[-1.08–1.37]
Age (years)	0.076	0.027	*0.004	[0.02–0.13]
Sex (F or M)	0.521	0.612	0.395	[-0.68–1.72]
BMI (kg/m²)	-0.017	0.07	0.809	[-0.15–0.12]
Disease duration (years)	0.001	0.604	0.904	[-1.18–1.31]

A multivariate logistic regression analysis was conducted to identify factors associated with sagittal trunk imbalance (U Group vs. N Group). The dependent variable was defined as sagittal imbalance (SVA > 50 mm), and the independent variables included trunk muscle mass, operative and non-operative leg muscle mass, age, sex, BMI, and disease duration on the operative side.

The results are presented in Table 5. The ROC analysis results are shown in Figure 3. Among the variables analyzed, age was found to be significantly associated with sagittal imbalance (β = 0.076, p = 0.004), indicating that older patients were more likely to exhibit a forward-shifted sagittal vertical axis. Trunk muscle mass, lower extremity muscle mass (operative and non-operative), sex, BMI, and disease duration were not significantly associated with sagittal imbalance in this model (Table 5).

## Discussion

SVA is a key indicator of trunk balance and quality of life and has been linked to decreased muscle strength in aging populations [1,17-20]. Evaluating whole-body alignment in the standing position using SVA is therefore essential for assessing adult spinal deformity and lower limb function.

While longer disease duration could contribute to postural deterioration, our data showed that age was the only variable significantly associated with spinal imbalance. This suggests age-related factors, rather than disease chronicity, BMI, or fall history, play a primary role in trunk balance impairment in THA patients.

Our study adds to the field by examining segmental muscle mass in a surgical OA population, underscoring the relevance of trunk and nonoperative limb muscle for spinal alignment. Although sarcopenia was not formally diagnosed, reduced trunk muscle mass was associated with poor SVA, implying localized age-related atrophy.

Clinically, this highlights the importance of evaluating muscle distribution and spinal-pelvic parameters in THA candidates. Targeted preoperative strengthening, particularly of the trunk and nonoperative limb, may support better postoperative outcomes.

Despite disease duration and pain severity often not correlating, patients typically seek THA once hip pain becomes severe. While BMI has been implicated in OA onset [21,22], our findings showed no relationship between BMI and trunk balance.

In our cohort, patients with poor balance (U group) were older than those in the N group, with no differences in BMI, disease duration, or falls. This supports aging as the primary factor for imbalance. Pelvic tilt was kyphotic in all postures, more pronounced in standing, and associated with poor trunk balance.

PI values, unique to individuals [23], did not differ between groups. However, since optimal alignment is achieved when LL approximates PI [24], the greater PI-LL mismatch in the U group suggests poorer balance [1,25]. While LL was not different, its value relative to PI was lower in the U group, indicating increased kyphosis.

Although TK showed no difference, thoracic alignment variability was likely masked by rib cage support. Prior studies suggested a trunk muscle mass threshold of 23 kg for maintaining alignment [26], but our cohort averaged 17 kg, likely due to age and OA. While 5.6 kg (nonoperative limb) and 17 kg (trunk) appeared sufficient to maintain alignment, the sensitivity and specificity were low, possibly due to obese or younger patients with poor SVA despite preserved muscle mass.

Offerski et al. [27] noted that in younger patients, imbalance may stem from hip anteversion transition, despite preserved muscle. Thus, differences in pelvic tilt or muscle mass may be masked by age distribution variations [4,5,17].

While SS, LL, and muscle mass differed between groups, regression analysis found only age significant, suggesting pelvic deformities were age-driven. This emphasizes the need for evaluating age-related deformities and muscle loss.

Lee et al. [28] reported that sarcopenic patients had higher PI-LL and PT but no SVA or TK differences. Similarly, in our cohort, sarcopenia was not directly linked to SVA or balance. Yet, trunk and nonoperative limb muscle mass was reduced, possibly due to sarcopenia’s multifactorial nature. As definitions of sarcopenia emphasize grip strength and gait speed, total limb/trunk muscle loss may go underrecognized, especially in older adults.

Hip arthritis affects pelvic tilt and contributes to lumbar kyphosis, leading to anterior tilt [27]. In elderly populations, posterior pelvic tilt worsens with OA onset [29]. Our study confirmed that patients with larger SVA had these age-related hip and pelvic changes. Muscle mass loss, especially in the trunk and nonoperative limb, was prominent in poor balance cases. Interestingly, operative-side muscle mass was less affected by aging or imbalance.

SVA-imbalanced patients demonstrated posterior pelvic tilt and lumbar kyphosis in standing. While THA alleviates operative-side pain, preoperative muscle loss can delay rehabilitation. Previous reports showed that strengthening external rotators and hip flexors/extensors improves function [8]. We also found nonoperative limb strengthening to be beneficial, supporting targeted preoperative rehabilitation.

This study has limitations. It was retrospective and cross-sectional, evaluating only preoperative patients and conducted in a single-center setting, which may introduce selection bias. Although SVA is a reproducible and practical X-ray measure, it is a static parameter that lacks insight into neuromuscular or dynamic aspects of posture. While we used a 50 mm cutoff [9], the low AUC of the ROC curve indicates limited clinical utility.

## Conclusions

In this cross-sectional study of preoperative THA patients, we found that spinal alignment, particularly SVA, was significantly associated with reductions in trunk and nonoperative lower extremity muscle mass. Patients with poor sagittal balance demonstrated age-related changes, including posterior pelvic tilt and lumbar kyphosis, which were not linked to sarcopenia by definition but were nonetheless characterized by localized muscle mass reduction. These findings suggest that muscle loss in patients with spinal imbalance may be overlooked if only whole-body sarcopenia criteria are applied. Furthermore, It is possible that bilateral muscle atrophy occurred due to disuse caused by osteoarthritis of the hip joint, but our results highlight the importance of evaluating spinal-pelvic alignment and muscle mass distribution in THA candidates to inform preoperative planning and optimize postoperative recovery.
